# The relationship between propagule pressure and establishment success in alien bird populations: a re-analysis of Moulton & Cropper (2019)

**DOI:** 10.7717/peerj.8766

**Published:** 2020-03-18

**Authors:** Tim M. Blackburn, Phillip Cassey, Julie L. Lockwood, Richard P. Duncan

**Affiliations:** 1 Centre for Biodiversity & Environment Research, University College London, London, United Kingdom; 2 Institute of Zoology, Zoological Society of London, London, United Kingdom; 3 Centre for Applied Conservation Science, and School of Biological Sciences, University of Adelaide, Adelaide, South Australia, Australia; 4 Department of Ecology, Evolution and Natural Resources, Rutgers University, New Brunswick, NJ, USA; 5 Institute for Applied Ecology, University of Canberra, Canberra, ACT, Australia

**Keywords:** Alien, Bird, Establishment, Propagule Pressure, Small Population Problem

## Abstract

A recent analysis by Moulton & Cropper (2019) of a global dataset on alien bird population introductions claims to find no evidence that establishment success is a function of the size of the founding population. Here, we re-analyse Moulton & Cropper’s data and show that this conclusion is based on flawed statistical methods—their data in fact confirm a strong positive relationship between founding population size and establishment success. We also refute several non-statistical arguments against the likelihood of such an effect presented by Moulton & Cropper. We conclude that a core tenet of population biology—that small populations are more prone to extinction—applies to alien populations beyond their native geographic range limits as much as to native populations within them.

## Introduction

A fundamental ecological tenet is that small populations with few individuals are more likely to go extinct than larger populations. This outcome arises because small populations are more susceptible to demographic, environmental and genetic accidents (i.e., stochasticity), and to Allee effects, that can cause them to die out, regardless of how suited they are to the environment they inhabit (Caughley & Gunn, 1996). This finding, termed the small population problem, is core to understanding the establishment success or failure of introduced alien populations (Lockwood, Hoopes & Marchetti, 2013). A plethora of research has consistently shown that introduced populations with small founding population sizes are more likely to go extinct (fail to establish) than those with large founding population sizes (see reviews in Cassey et al., 2005; Lockwood, Cassey & Blackburn, 2005; Colautti, Grigorovich & MacIsaac, 2006; Hayes & Barry, 2008; Simberloff, 2009; Cassey et al., 2018). This outcome emerges from historical (Veltman, Nee & Crawley, 1996) and experimental studies (Memmott et al., 2005); for taxa as diverse as plants (Zenni & Simberloff, 2013), insects (Rossinelli & Bacher, 2015), birds (Brook, 2004) and mammals (Forsyth & Duncan, 2001); on islands (Duncan, 1997), continents (Marchetti, Moyle & Levine, 2004), and worldwide (Sol et al., 2012). The relationship between founding population size (often termed propagule pressure; Lockwood, Cassey & Blackburn, 2005) and establishment success is sufficiently general that it is considered a “null model for biological invasions” (Colautti, Grigorovich & MacIsaac, 2006).

However, this core precept of invasion science has been questioned in a series of papers by M. Moulton and colleagues. Most recently, Moulton & Cropper (2019) critiqued the assumptions underlying the role of propagule pressure as a driver of establishment success and the data used to support this relationship. The authors cited evidence that they purported strongly supports other explanations for why introductions of alien species fail, and presented two broad analyses of a published dataset of bird introductions (Sol et al., 2012) that they claimed do not support a relationship.

These analyses are potentially the most important part of Moulton & Cropper’s paper as they challenge not only the findings of the vast majority of the invasion biology literature (Simberloff, 2009; Cassey et al., 2018), but also previous studies based on this specific dataset (Sol et al., 2012; Duncan et al., 2014). In the first analysis, Moulton & Cropper used only introduction events that involved between 2 and 10 individual birds being introduced (roughly 27% of the dataset), and showed that establishment success of these nascent populations does not vary across this range. In the second, they analysed the full data set by grouping the data into 38 propagule pressure categories, calculating establishment probability for each category (their Table 2), and then regressing these establishment probabilities against the propagule pressure values for each category. They claim that this analysis also shows that propagule pressure is not related to establishment success in these bird introductions.

Here, we revisit the Sol et al. (2012) data, and the analyses presented by Moulton & Cropper (2019), to explore whether they fail to show the propagule pressure effect, as Moulton & Cropper claim.

## Methods

The dataset published by Sol et al. (2012) includes information on a wide variety of traits that might affect establishment success, but we focus on the subset of 832 separate introductions of bird populations worldwide analysed by Moulton & Cropper (2019), and presented by them as an appendix to their paper. This subset includes information on taxonomic order, family and species, group (four categories, though it is not stated to what these groups refer), propagule pressure (number of individuals introduced), and introduction outcome (0 = failure, 1 = success). To this dataset we added information on region of introduction (Australasia, Ethiopian, Nearctic, Neotropical, Oriental, or Palaearctic) and location of introduction (a total of 46 different country or island territory locations, nested within region) from the original source (Sol et al., 2012). Moulton & Cropper (2019) updated the taxonomy used by Sol et al. (2012), and we use their revised groups.

Moulton & Cropper present two analyses of the propagule pressure effect. We do not consider the first of these as it involves selecting a small subset of the data and discarding 73% of the information (we return to this issue in the Discussion). We re-analysed the data used in the second analysis (their Table 2). In this analysis, Moulton & Cropper grouped introductions into 38 categories based on the number of individuals introduced, with each category containing very different numbers of data points (ranging from 4 to 227; the rationale for choosing these 38 categories is not described). Due to the different numbers of data points, the uncertainties associated with estimates of establishment probability for each category will differ. Figure 2 in Moulton & Cropper shows these uncertainties as 95% confidence intervals, but these appear to have been calculated incorrectly. We recalculated the confidence intervals associated with the probability of establishment for each category using Wilson’s method for binomial data (Brown, Cai & DasGupta, 2001). For the first category, with 64 successes and 163 failures, this gives an estimated establishment probability = 0.282, with 95% confidence intervals of 0.227 to 0.344. Figure 2 in Mouton & Cropper is misleading in showing this confidence interval ranging from below 0 to almost 1.

A robust way to analyse the data in Table 2 of Moulton and Cropper is to fit a model assuming binomial outcomes, estimating the probability associated with obtaining the observed number of successes given the number of introductions in each category, with founding population size for each category included as an explanatory variable (log10 transformed). We perform this analysis on the data presented in Table 2 of Moulton & Cropper (but correcting the mid-point of the 38th category, for which Moulton & Cropper reported the minimum founding population size; this change does not affect our conclusions), using the R code presented in the Appendix. This analysis uses the aggregated categories that Moulton & Cropper argue for, but correctly weights the analysis by accounting for the different number of data points in each category.

We can obtain precisely the same result by converting the data in Table 2 of Moulton and Cropper to a series of 0/1 outcomes for each of the 832 introductions and then analysing these binary responses using logistic regression (see Appendix). This highlights that if the analysis is done correctly, it makes little difference whether or how the data are aggregated, which undermines Moulton and Cropper’s point that aggregating the data is somehow necessary. Aggregation does, however, lose important information because we have to use the average value for propagule pressure in each category rather than using the actual propagule pressure values associated with each introduction.

A further advantage of analysing the data as binary outcomes is that we can include other covariates related to individual introductions that might explain establishment success. As noted by Moulton & Cropper, taxa and regions may differ in founding population sizes, and in exactly how founding population size influences establishment success. We therefore used a Generalised Linear Mixed Model (GLMM) with binary response data to estimate the relationship between establishment probability and founding population size, including random effects for bird order and family (nested within order), geographical region (the six regions identified by Sol et al. (2012) for these data) and location (the 46 locations identified by Sol et al. (2012) nested within geographical region), using the glmer function in the lme4 package v. 1.1-21 (Bates et al., 2015) in R v. 3.6.1 (R Core Team, 2019). The random effect for geographical region did not improve the model, and so was excluded. We additionally used the package MuMIn (Bartoń, 2019) to calculate the variance explained by the GLMM, using the method for binomial models described by Nakagawa, Johnson & Schielzeth (2017). The R code for these analyses is presented in the Appendix.

## Results

**Figure 1:**
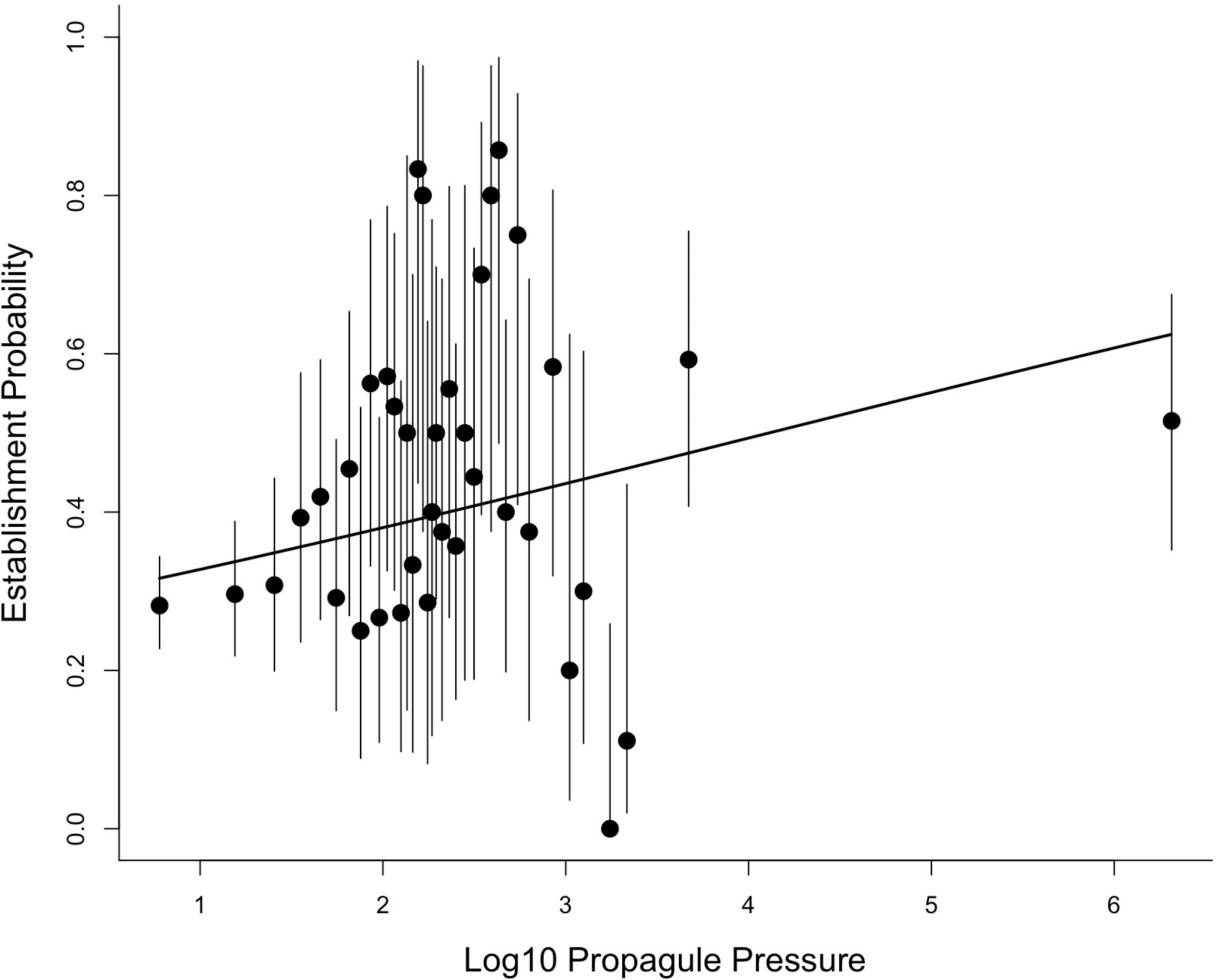
The relationship between establishment probability and log10 propagule pressure for the 38 categories reported by Moulton & Cropper for the introduction events in the Sol et al. (2012) database. This figure corrects errors in the 95% confidence intervals (shown as bars), and in the midpoint of the final category in the version originally published by Moulton & Cropper (2019).

Reanalysing the aggregated data in Table 2 of Moulton & Cropper reveals a highly significant relationship between establishment probability and founding population size (slope (on the logit scale) = 0.23, standard error (s.e.) = 0.06, z-score = 3.9, *P* < 0.0002). Figure 1 shows this relationship, and the uncertainties around the estimated establishment probabilities for each category calculated using the appropriate standard error equation (see Methods).

**Figure 2:**
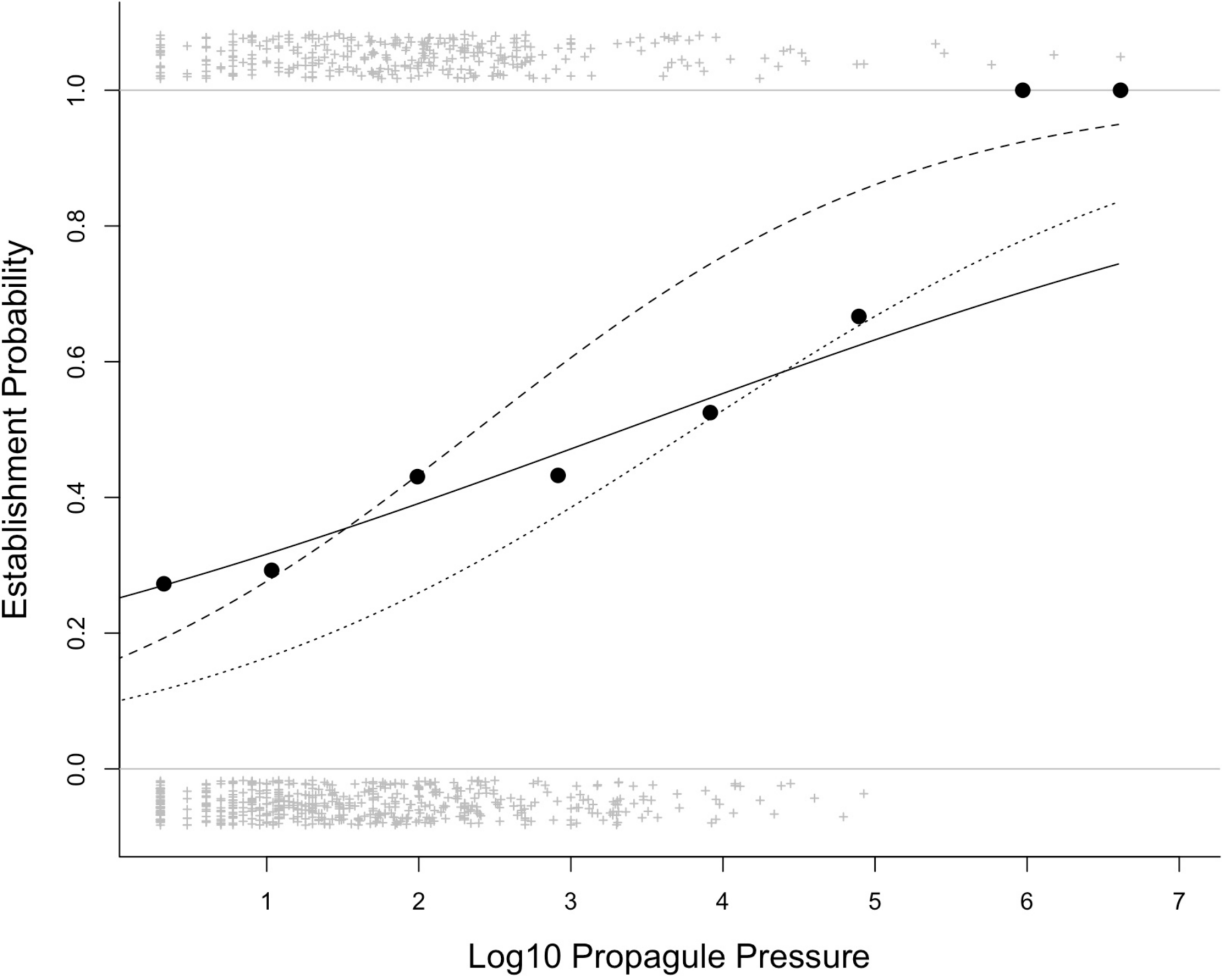
The relationship between establishment probability and log10 propagule pressure for the 832 introduction events in the Sol et al. (2012) database. The lines show the model fit of a simple logistic regression (solid line) and a General Linear Mixed Model with random effects of order, family (nested within order) and location (dashed line). The dotted line shows the fit of a simple logistic regression for Galliformes only (lower probability of establishment success; estimate ± s.e. = 0.581 ± 0.106, *z* = 5.5, *n* = 404, *P* < 0.0001); establishment probability and log10 propagule pressure are still significantly positively related with Galliformes excluded (GLMM with family and location as random effects: estimate ± s.e. = 1.21 ± 0.22, *z* = 5.6, *P* < 0.0001). Crosses show the outcomes (success, failure) of the introduction events, while the filled circles show the mean establishment probability for all species for events lumped into eight classes of equal bin-width.

A simple logistic regression shows a strong positive relationship between establishment probability and founding population size across the 832 introduction events in the data used by Moulton & Cropper (slope ± s.e. = 0.33 ± 0.07, *z*-score = 4.6, *P* < 0.0001; Fig. 2). Incorporating information on taxonomy and location of introduction in a GLMM strengthens the effect of founding population size (slope ± s.e. = 0.96 ± 0.11, *z*-score = 8.4, *P* < 0.0001, marginal pseudo-*R*^2^ = 0.125; conditional pseudo-*R*^2^ = 0.480; Fig. 2).

## Discussion

Exceptional claims require exceptional evidence. Against a backdrop where the large majority of statistically robust studies find a significant positive relationship between founding population size and establishment success for introduced alien populations (see the meta-analysis by Cassey et al., 2018), Moulton & Cropper’s (2019) claim that this does not hold for a large sample of bird introductions appears exceptional, particularly given their results are in stark contrast to previous analyses of the same data (Sol et al., 2012; Duncan et al., 2014). This discrepancy motivated our re-analysis of the data. We show that when the data are analysed correctly there is a strong relationship between founding population size and establishment success, an outcome that is robust to whether the data are aggregated by propagule size or not.

How then do Moulton & Cropper fail to find the propagule pressure effect in their analyses? The simple reason is that their analyses are flawed. The first analysis (which we did not reanalyse) uses only 27% of the data covering a narrow range of propagule sizes (2 to 10 individuals). It is not surprising that there is no propagule pressure effect across such a narrow range of founding population sizes. It is common for establishment success to be a sigmoidal function of the logarithm of founding population size (see e.g., figure 3 in Cassey et al., 2018), as is the case here (Fig. 2), such that success is consistently low across very low founding population sizes. Restricting analysis to the lower tail of this S-shaped curve fails on both statistical and rational grounds. Moulton & Cropper give no logical reason for selecting 10 individuals as the upper population size for this analysis. One such criterion might be 49 individuals, as the IUCN Red List classifies species with populations of <50 as Critically Endangered under Criterion D, and therefore facing an extremely high risk of extinction. We would note that propagule pressure explains significant variation in establishment success (i.e., the inverse of extinction probability) within the range 2 –49 individuals (GLMM using the methodology as described for Fig. 2: slope ± s.e. = 0.89 ± 0.31, z-score = 2.8, *P* < 0.01). Nevertheless, we do not advocate this approach, as there is no magic population size above which we expect stochastic effects to disappear (Traill et al., 2010; Brook et al., 2011), and no good reason to restrict analysis to arbitrary subsets. The essence of the propagule pressure effect is that small populations are more susceptible to extinction than large populations. The appropriate comparison is thus one where outcomes for small founding population sizes are compared to larger ones, not to restrict analysis to very small populations, which we know are likely all to have a low establishment probability.

Moulton & Cropper’s (2019) analysis based on all of the data they take from Sol et al. (2012) is flawed for different reasons. Moulton and Cropper argue that these data suffer from a bias due to the aggregation of releases. Propagule pressure is typically defined as the total number of individuals of a species introduced at a given location (Blackburn, Lockwood & Cassey, 2009). However, these individuals are not necessarily released all in one go such that many of the introduction events in the Sol et al. (2012) dataset include releases of the same species at similar locations but at different times. If early success in establishment prompts further introductions, then aggregating over multiple introductions might overestimate founding population size for successful introductions, but not for unsuccessful ones, creating a spurious propagule pressure effect. Separating pre- from post-establishment releases is important in quantifying propagule pressure, but previous analysis of historical data has shown that success in establishment does not consistently influence future introductions (Blackburn et al., 2013), implying that this effect is unimportant. Moreover, as we have shown elsewhere, founding population size predicts establishment success when birds are liberated in a single release, and when controlling for the number of releases in situations with known multiple releases (Blackburn et al., 2013).

Even if the aggregation of multiple releases were a problem, aggregating the data into propagule size bins does not alleviate this. It is difficult to tell exactly how Moulton & Cropper analysed the binned data (they mention both weighted regression with normal errors and logistic regression, but with scant methodological details). Certainly, their calculation of the confidence intervals associated with these bins was incorrect. We have shown that analysing the binned data assuming binomial outcomes, which correctly accounts for the different number of data points in each category, reveals a highly significant relationship between propagule pressure and establishment success (Fig. 1). We also show (see Appendix) that this result is robust to how the data are aggregated: aggregating using the 38 bins (as Moulton and Cropper advocate) is statistically identical to analysing the data as binary outcomes (no aggregation) if we use the average value for propagule pressure in each category. Moreover, analysing the data as binary outcomes and including additional covariates strengthens the propagule pressure effect (Fig. 2). Thus, Moulton & Cropper’s argument that propagule pressure effects are a spurious consequence of data artefacts is not supported when the data are analysed correctly. Their arguments are further refuted by the results of experimental studies that are not subject to these artefacts, and which consistently demonstrate the same propagule pressure effect as observed in historical data (Cassey et al., 2018).

The standard statistical approach to testing for the propagule pressure effect is logistic regression, with establishment success as a binary outcome, as this allows introduction-level covariates, including propagule pressure, to be included in the analysis. Analysing the data this way reveals a strong positive relationship between founding population size and establishment success. Moulton & Cropper evidently applied this method, but expressed “serious reservations about doing this analysis on individual releases for such a diverse set of species, locations and times”, and did not present any results for this. The issue that Moulton & Cropper raise about species and locations is a valid one, as multiple introductions of the same species or multiple introductions to the same location may suffer from the problem of non-independence, or taxa and regions may systematically differ in founding population size, and in exactly how founding population size influences establishment success. Moulton & Cropper report taxonomic and regional differences in establishment success and founding population size (albeit without supporting statistics) as if this was novel, although these effects are well-known (Leung & Mandrak, 2007; Leung et al., 2012) and have been demonstrated quantitatively using bird introduction data (see e.g., Cassey et al., 2004; Blackburn, Lockwood & Cassey, 2009). For example, game birds tend to be introduced in large numbers but also to have lower overall establishment probability for a given founding population size (Fig. 2). Fortunately, statistical methods exist that allow us to incorporate taxonomic and regional effects, and using a GLMM we show that the propagule pressure effect is robust to the diversity of species and locations in the data (Fig. 2). Indeed, incorporating this extra information strengthens the propagule pressure effect, because the random effects help to partition the taxonomic and regional variation in establishment probability.

Besides their statistical analyses, other arguments raised by Moulton & Cropper (2019) against the propagule pressure effect are also straightforward to refute. For example, they argue that most studies that show the effect rely on historical data, and these data inevitably contain inaccuracies. All data are subject to measurement error, but Moulton & Cropper do not explain why such errors should produce a systematic bias toward a propagule pressure effect. Moulton & Cropper focus on uncertainties in historical data on bird introductions, but no such uncertainties occur in the numerous experimental studies (e.g., Memmott et al., 2005) that consistently show strong propagule pressure effects that are statistically indistinguishable from those in historical data (Cassey et al., 2018).

Moulton & Cropper (2019) further argue that invasion biologists champion the propagule pressure effect “despite numerous studies that show equally important roles for location-level factors…and species-level factors”. In fact, invasion biologists routinely recognise that species and location-level factors, along with propagule pressure, influence introduction outcomes, and we can point to our own work in this regard (see e.g., Duncan, Blackburn & Sol, 2003; Lockwood, Hoopes & Marchetti, 2013; Duncan et al., 2014; Duncan, 2016; Redding et al., 2019). Which determinant of establishment success dominates can depend on context (Duncan, 2016), but most published research that includes a metric of propagule pressure shows that it is consistently the strongest factor of the set analysed. Our recent work (Redding et al., 2019), cited by Moulton & Cropper, demonstrates this: a global analysis of establishment success in birds, including data for multiple species and location-level characteristics, and propagule pressure, showed that location-level characteristics together explained the most variation in establishment success worldwide. However, propagule pressure was the single variable that explained the most variation in the analysis (Redding et al., 2019). This research built on our earlier work on the same data as analysed here, where we used theoretical population models to demonstrate that while founding population size is important, a major cause of establishment failure was introduction to unfavourable locations where establishment probability was inherently low. Even large founding populations cannot overcome such environmental mis-matches (Duncan et al., 2014). This mechanism explains the wide confidence intervals in Fig. 1—variation that we have shown can be explained in turn by location and species-level effects (Duncan et al., 2014; Redding et al., 2019). It is simply incorrect to conclude, as Moulton & Cropper do, that we argue that “introductions fail mostly because insufficient numbers of individuals were released”. Nevertheless, the observation that other factors affect establishment success does not alter the outcome that propagule pressure consistently has a strong, independent, positive effect on establishment.

Finally, Moulton & Cropper argue that “at any arbitrarily small size, natural populations that are declining are very different from introduced populations that are increasing.” Increasing and decreasing populations are indeed likely to differ given their distinctive population trajectories. But not all introduced populations increase: the majority decline to extinction. This observation highlights the similarity between alien and native populations, and emphasises that the fundamental processes are the same. The small population problem applies to all small populations, regardless of the growth trajectory or conservation status of the species to which they belong. Demographic, environmental and genetic stochasticity, and Allee effects, do not know whether a population is native or alien.

In summary, we argue that Moulton & Cropper’s conclusion that there is no relationship between propagule pressure and establishment success in alien bird populations rests entirely on flawed analyses. The well-known fact that features other than propagule pressure explain variation in establishment success is not relevant to the empirical outcome: a correct analysis of the data shows a highly significant relationship. Their argument that propagule pressure effects are a spurious consequence of data artefacts is not supported by a now-large body of published research, and it ignores the results of experimental studies that are not subject to these artefacts. Furthermore, their argument runs counter to core tenets of population biology and the wide-ranging support for the link between population size and extinction risk. This link does not mean that all small populations necessarily go extinct, nor that all large populations persist, because the processes that cause the small population problem are stochastic, not deterministic—plus, as we have repeatedly noted and demonstrated (Duncan, Blackburn & Sol, 2003; Blackburn, Lockwood & Cassey, 2009; Lockwood, Hoopes & Marchetti, 2013; Duncan, 2016; Redding et al., 2019), features of the species and environments matter to establishment success of alien species too. The core tenets of population biology nevertheless apply to populations beyond their native geographic range limits as much as they do to populations within them, as has been shown repeatedly and consistently in field and natural experiments for a range of taxa, including the data analysed here.

##  Supplemental Information

10.7717/peerj.8766/supp-1 Supplemental Information S1 Data for Fig. 1 and associated analyses

10.7717/peerj.8766/supp-2 Supplemental Information 2 Data for Fig. 2 and associated analyses

10.7717/peerj.8766/supp-3 Supplemental Information 3 R code for the figures and analysis

## Additional Information and Declarations

### Data Availability

The following information was supplied regarding data availability: Data and code are available in the Supplemental Files.
